# Surgical site infection leading to gangrene and amputation after ambulatory surgical care of an ingrown toenail: a case report

**DOI:** 10.1186/s13037-019-0225-1

**Published:** 2019-12-16

**Authors:** Aimé Gilbert Mbonda Noula, Joel Noutakdie Tochie, Landry W. Tchuenkam, Desmond Aji Abang, René Essomba

**Affiliations:** 1 Department of Orthopedic Surgery, National Social Insurance Fund Hospital, Yaoundé, Cameroon; 2 Department of Anaesthesiology and Critical Care Medicine, Faculty of Medicine and Biomedical Sciences, University of Yaoundé I, Yaoundé, Cameroon; 3 Department of Surgery and sub-Specialties, Faculty of Medicine and Biomedical Sciences, University of Yaoundé I, Yaoundé, Cameroon; 4 Global Health System Solutions (GHSS) and Faculty of Sciences, University of Buea, Buea, Cameroon; 5 Higher Institute of Medical Technology, Yaoundé, Cameroon

**Keywords:** Ingrown toenail, Nail surgery, Gangrene, Ambulatory surgery

## Abstract

**Background:**

Currently, the management of ingrown toenail (onychocryptosis) ranges from conservative medical management to surgical treatment. Surgical management is typically performed as an outpatient procedure due to it numerous advantages such as the simplicity of the technique and the low incidence of postoperative complications. The most common postoperative complications are recurrences and surgical site infections, whereas gangrene complicating a surgical site infection has been scarcely reported. We are reporting a rare complication following ambulatory surgery untimely requiring amputation.

**Case presentation:**

A twelve-year-old boy was referred to our orthopedic surgical department for a surgical site infection complicating an initial surgical management of a left ingrown big toenail leading to a dry gangrene of the affected toe. The gangrene toe was amputated under peripheral nerve block and the patient was discharged home the same day on antibiotics, analgesics and with sessions of rehabilitation and psychological support planned. The postoperative course was uneventful at 6 months of follow-up.

**Conclusion:**

The authors report this case to draw clinicians’ attention, especially wound care specialists, orthopedists and podiatrists to this rare but potentially debilitating disease.

## Background

The nail may be subject to various congenital or acquired pathologies. The inesthetic nature and symptoms associated with these disorders generally lead the patients to consult. Pain is one of the major symptoms contributing to the consultation of patients suffering from nail diseases, particularly ingrown toenails (onychocryptosis) [1]. Some of these nail diseases can have a significant impact on the lives of patients, both psychologically and physically [2]. The ingrown nail results from an imbalance between the width of the nail, a hypertrophy of the plate (spicule) which pierces the lateral fold of the nail and penetrates the skin, causing a subsequent inflammatory reaction to the foreign body and a secondary infection [3]. A 10-year study conducted in Korea found an increase in the incidence of ingrown nails from 360 cases per 100,000 persons in 2004 to 462.2 cases per 100,000 persons in 2013 [4]. This demonstrates the rapid time trend of this pathology. Ingrown nail affects women more than men [3]. Cho et al reported a peak in young people and adults aged above 50 years. The ingrown toenail often affects the big toe because it is generally caused by repeated mechanical trauma exerted on the flat surface of the nail by shoes, especially tight-fitting ones [5]. Ingrown toenails can be classified into into four types namely the childhood type, the lateral and distal hypertrophy of the fold of the nail, pincer nails, and the juvenile ingrown toenail [6]. In 2008, Kline et al [7] introduced a simple classification system associated with an algorithm for its management. This system classifies the ingrown nail into five types and has the advantage of stratifying the severity of onychocryptosis. The therapeutic approaches may be surgical or non-surgical depending on the severity of the lesion and clinicil assessment [7]. In general, the surgical approach is recommended when non-surgical treatment and physical measures such as wearing open-toed shoes have failed. Currently, it is recommended to perform surgical management of ingrown toenails as an outpatient or ambulatory procedure under perpheral nerve block using local anesthetic drugs [8]. The complications during or after this surgical procedure are rare [8]. A recent study reported that the rate of complications observed in children found 12–37% for recurrences, 8% for soft tissue infections and 1–2% for osteomyelitis [9].

A search of PUBMED and EMBASE between inception to July 2019 with keywords: “ingrown” “toenail” “nail disease” “nail surgery” “lidocaine” “epinephrine” and “outpatient surgery”, revealed few systematic reviews, observational studies or clinical trials. The published data retrieved from the database search was carried out in developed countries and none included ambulatory procedures. We are report a case of a 12-year-old boy presenting with a rare complication of ingrown toenail following ambulatory surgery untimely requiring amputation.

## Case presentation

A 12-year-old Cameroonian male adolescent with an uneventful past medical, family and psychosocial histories was referred to the orthopedic department of the National Social Insurance Hospital in Yaoundé, Cameroon for a surgical site infection of a left big toenail leading to a dry gangrene of the affected toe following surgical treatment for an ingrown toenail 36 h before his current hospital presentation.

**Fig. 1:**
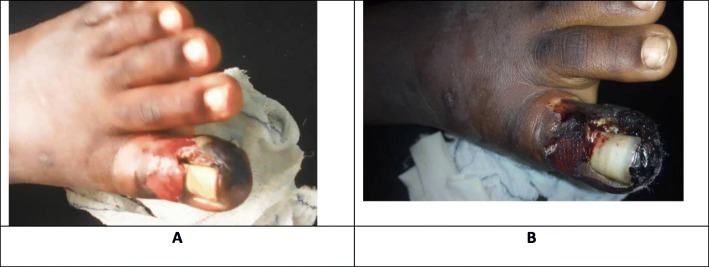
First postoperative day demonstrating superficial necrosis of the left big toe (**a**: 12 h postoperation **b**: 24 h postoperation)

The patient had been suffering from an ingrown nail of the hallux of the left foot for several months. The pain was aggravated by wearing closed shoes and was unremitting to usual analgesics such as paracetamol and diclofenac. Persistent pain caused by the ingrown nail prompted the consult of a general practitioner who proposed a surgical treatment for the patient. His preoperative workups included a complete blood count, prothrombine time, partial thromboplastine time, fasting blood glucose and HIV tests which were all normal. The surgical procedure was performed under local anesthesia containing lidocaine 2% and epinephrine. Intravenous Amoxicilline 1 g and metronidazole 500 mg were administered 30 min before the onset of the surgical procedure. An annular digital block was tied round the necrosed toe. Local anesthesia was infiltrated at the postero-lateral aspect and base of the affected toe. The FROST surgical technique was performed by the general practitioner. The technique entailed an “L-shaped” soft tissue dissection to expose and excise the root of the nail and any abnormal soft tissue associated with it [1]. There was no intraoperative complication and the patient was discharged home an hour later with an antibiotic regimen consisting of oral amoxicilline 1 g/8 h and metronidazole 500 mg/8 h. The patient returned a day after the operation, with complains of severe pain of the operated toe. He was administered paracetamol and codeine and the wound was dressed (Fig. 1). Findings during the dressing were; a very tender amputated toe stump with a black plaque covering 40% of the medial side of the left big toe. A wet dressing of the toe was done using normal saline and the patient was sent back home. On the second postoperative day the persistence of pain and the enlargement of the black plaque into necrosis of the entire toe motivated the referral of the patient to the orthopedic surgical department of the National Social Insurance Hospital in Yaoundé, Cameroon.

**Fig. 2:**
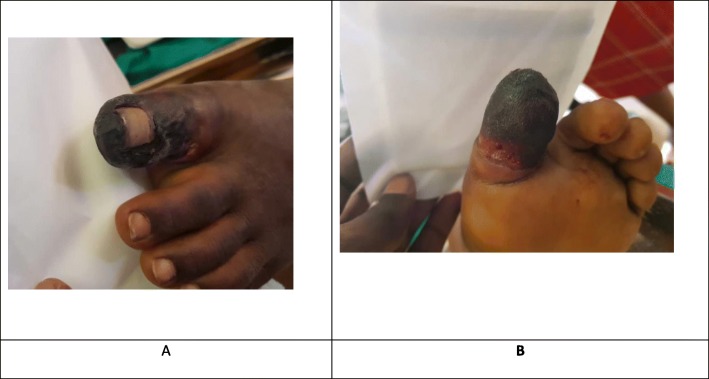
Second postoperative day. **a**: dorsal view of the toes of the left foot; **b**: plantar view of the toes of the left foot

**Fig. 3:**
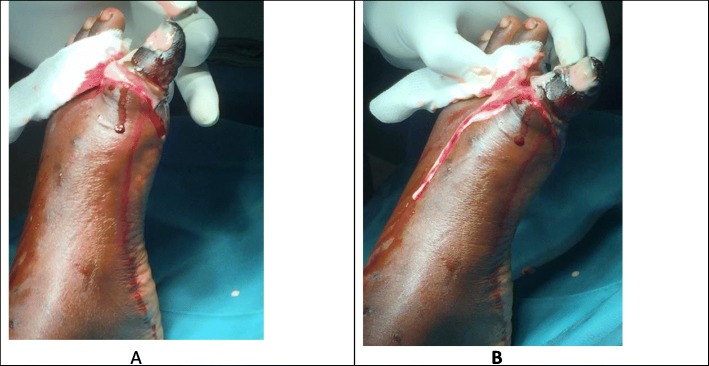
**a** and **b:** Intrao-perative findings during the surgery for amputation of the left big toe

We received the patient in severe pain rated 8/10 using the visual analogue scale. He was overweight with a body mass index (BMI) of 28.89 kg/m^2^ all his vital signs were normal. Local examination of the affected toe showed a necrosed (black in colour) and dried left big toe with a loss of sensation (Fig. 2). The left big toe also portrayed signs of inflammation such as being swollen, hot and very tender. All distal pulsations were present and of good volume. There were no swollen lymph nodes on examination of both lower limbs. In view of these findings, we made the diagnosis of dry gangrene of the left big toe. After informing the patient’s family members about the pathology and obtaining consent from his parents, we planned a surgical debridement of the necrosed toe. Another laboratory panel performed before the debridement showed C-reactive proteins (CRP) at 96 mg, hemoglobin at 15 g/dl and leucocytosis at 12,000/mm^3^. Surgical debridement of the necrosed toe was done under a combined saphenous and popliteal nerve block using ropivacaine. Intraoperative findings were necrotic tissues of the entire left big toe (Fig. 3). After explanation of the findings to the parents and obtaining a second parental consent, we opted for total amputation of the left big toe rather than surgical debridement. No intraoperative complication occurred. Postoperatively, his treatment consisted of oral ofloxacin 200 mg/12 h, paracetamol 1 g/6 h and tramadol 50 mg/8 h. He was discharged home the same day of the surgery, precisely 11 h after the surgery when the sensitive and motor block induced by the peripheral nerve blocks had waned. Walking physiotherapy sessions were done from the fifth postoperative day to quickly rehabilitate the patient. We equally recommended psychological support by a psychologist due to the trauma associated with the amputated toe. His follow-up was uneventful at 6 months following the ambulatory surgical amputation of his left big toe.

## Discussion

The nail is composed of the nail matrix, the nail bed, the hyponychium, the lateral (paraonychium) and proximal (eponychium) folds [10]. The role of eponychium and paraonychium is to protect the nail bed and guide its growth. However, if the lateral edges are hypertrophied, they can cause ingrown toenails. The vascular system of the toe is anastomotic. Two arteries give capillaries that run obliquely into the proximal fold of the nail and the nail matrix and parallel to the surface of the nail bed [11]. The innervation varies according to the toes [10], however the digital peripheral nerves will be divided in three branches innervating the bed of the nail, the digital tip and the pulp [10].

The incarnated nail then results from an anatomical imbalance between the different constituent elements of the nail [3]. According to Minotto et al there are four large anatomical presentations of the ingrown nail. The first is the incarnated nail in infancy that can present itself as [12]:

- The pulp interlocking with the nail of the normally directed nail.
- Congenital hypertrophic paraonychium of the hallux; mainly the medial fold which can cover up to half of the nail.
- Congenital dystrophy of the big fingernail.

The second entity is the juvenile incarnated nail which according to the presentation can be classified in three stages [6]:

- Stage 1 marked by erythema, edema and pressure pain.
- Stage 2 marked by infection and purulent flow.
- Stage 3 marked by purulent granulation tissues and hypertrophy of the paraonychium.

The third is the ingrown toenail in the form of pliers, we find in this presentation, a bilateral and symmetrical curvature associated with a lateral deviation of the long axis of the bed of the nail [3]. The fourth is a hypertrophy of the folds of the lateral and medial nail (paraonychium) [12]. Our case presented a juvenile ingrown toenail nail of the medial fold. In view of his age and complaints, the general practitioner opted for a surgical management. The primum movens of this pathology is the reaction to a foreign body. This is due to the presence of keratinous material in the flesh of the toe which causes an inflammatory response (redness, pain, edema and heat). Such a reaction leads to major psychosocial consequences [3].

Patients with ingrown toenails complain of painful, swollen and tender nails with or without purulent discharge. It is always important to exclude underlying conditions such as diabetes or arterial insufficiency. The indexed patient did not present any of these diseases as evident by his unremarkable past history.

Several authors have tried to classify ingrown toenails based on their clinical presentations, the severity of the lesions, and the appropriate treatment. In 2002, Mozena [13] attempted to classify ingrown toenails (Steps I, IIa, IIb, III). Then, in 2007, Martinez-Nova et al [14] also proposed a new classification (Stages I, IIa, IIb, III, IV). But it is in 2008 that Kline et al [7] provided the most universally adopted classification used till present date [4]. Using this classification, this pathology can be classified into four stages (Table 1) [7]. The postoperative outcome of the patient depends on his stage.

**Table 1:** Therapeutic options according to classification of toe ingrowth [7]

Onychrocryptosis (toe ingrowth)				
Stage I	Stage II	Stage III	Stage IV	Stage V
Local irritation No infection, pus or granulation tissue.	Infection without a history of onychocryptosis. Pus and/or Granulation tissue	Infection with history of more than one episode of onychocryptosis. Pus/Granulation	Infective onychocryptosis with partial onycholysis of a single nail border.	Infective onychocryptosis with partial onycholysis of both nail borders.
May Perform Cauterization			Do Not Perform Cauterization	
Treatment (Stage 1)	Treatment (Stage 2)	Treatment (Stage 3)	Treatment (Stages 4,5)	
1.Self Treatment 2. Slant Back 3. Suppan 4. Chemical 5. Laser 6. Cold steel 7. Daily soaking	1. Slant Back 2. Suppan 3. Chemical 4. Laser 5. Cold steel 6. Daily soaking	1. Winograd 2. Chemical 3. Laser 4. Cold steel 5. Daily Soaking	1. Remove offending nailborder or nail plate. 2. Resection of hypertrophic ungualabia. 3. Radiographs or magnetic resonance imaging is indicated in Chronic cases to rule out osteomyelitis. 4. Address osteomyelitis or periostitis. 4. Return for secondary stage procedure to include matrixectomy. 5. Resolve infection to return to Stage 1,2 or 3 for appropriate procedure.	

Ropivacaine appears to be the local anesthetic drug preferred by surgeons and anaesthetists for peripheral nerve block [3] in nail surgery because of its rapid onset of action and long duration of action of analgesic effects of up to 9 hours. However, because lidocaine is cheaper and readily available in low-resource setting, it is more used combined with epinephrine as adjuvant for the anesthesia of this surgery [5]. It is worth to mention that adrenaline as an adjuvant to local anesthesia also potentiates the local anesthetic effects. Denkler in 2001 [15] in a review showed that the use of lidocaine associated with epinephrine in ingrown nail surgery presented a risk of 1 case per 100,000 persons and was safe. The combination of epinephrine, which is a strong alpha and beta receptor agonist, has the advantage of local vasoconstriction, reduced bleeding and prolonged anesthesia. However, this association remains controversial in the surgery of the fingers and toes. Indeed, its strong vasoconstrictor action becomes dangerous because of the risk of vasospasm in the terminal arterioles of the peripheral tissues; this can potentially lead to ischemia [16] and subsequent gangrene [17]. However, toenail gangrene can also be found when an excessive volume of local anesthetics is injected circumferentially around the toe [8]. In the contemporary literature, very few good-quality studies have shown a correlation between the use of epinephrine as an adjuvant to local anesthesia and the occurrence of toenail gangrene [18, 19]. However, the use of epinephrine should be avoided in patients with peripheral cardiovascular diseases or increased risks of peripheral ischemia such as Raynaud’s syndrome due to the vaso-constricting effects of epinephrine which may aggravate ischemia [20, 21]. Complications of peripheral nerve blocks such as intravascular injection of the local anesthetic drug are not uncommon even under ultrasound-guided administration of the local anesthetic drug [22, 23]. However only a large amount of local anaesthesia with a maximum of 75mg can cause complications [24, 25]. In the present case we did not encounter this complication, despite blind administration of the local anesthetics. Hence, re-inforcing the feasibility of ambulatory surgery and to re-iterate frequent management challenges of dermatological disorders encountered in resource-limited settings [26–28]. Although the definitive surgical management by amputation of the dry gangrene toe was necessary, it is important to mention that some authors advocate auto-amputation of dry gangrenous digits in resource-limited settings where no surgeon is available [29, 30].

The limitation of this case report is the fact that it is not clear whether the cause of the gangrene was the due to the use of lidocaine combined with epinephrine by the general practitioner for local anesthesia. The rapid onset of the gangrene may be due to the used of the use of lidocaine combined with epinephrine and/or an underlying undiagnosed peripheral arterial disease.

## Conclusion

The surgical management of a dry gangrene of a big toe via an ambulatory procedure seems relatively simple with rare severe complications, especially when the intervention is performed by inexperience health personnel. Hence, this case report seeks to draw clinicians’ attention, especially wound care specialists, orthopedists and podiatrists to this rare but potentially debilitating complication, toe amputation.

## Data Availability

Data sharing is not applicable to this article as no datasets were generated or analyzed during the current study.

## Abbreviations

BMI: Body mass Index
CRP: C reactive protein
HIV: Human immunodeficiency virus
